# Small RNA-mediated regulation of cross-kingdom gene expression in sugar beet genotypes resistant and susceptible to rhizomania

**DOI:** 10.1099/jgv.0.002193

**Published:** 2025-12-17

**Authors:** Evan M. Long, Rajtilak Majumdar, Carl A. Strausbaugh, Imad A. Eujayl

**Affiliations:** 1 Northwest Irrigation and Soils Research Laboratory, United States Department of Agriculture - Agricultural Research Service, Kimberly, ID, 83341, USA

**Keywords:** beet necrotic yellow vein virus, KEMS12, microRNA (miRNA), rhizomania, small non-coding RNA, sugar beet

## Abstract

Rhizomania in sugar beet causes significant yield and sucrose loss worldwide. The disease is caused by Beet necrotic yellow vein virus (BNYVV) and vectored by the plasmodiophorid, *Polymyxa betae*. Resistance to rhizomania in commercial cultivars is currently dependent upon the use of *Rz1* and *Rz2* resistant genes in sugar beet. We have developed an ethyl methanesulphonate mutant breeding line (KEMS12; PI672570) that is highly resistant to rhizomania. Using rhizomania-resistant (R) and susceptible (S) sugar beet breeding lines, natural infection and comprehensive RNA sequencing, we have identified the accumulation of a unique set of small non-coding RNAs (sncRNAs) derived from both the sugar beet plant and the BNYVV virus during active infection that may have possible regulatory roles in the resistance and/or susceptibility to rhizomania. Examples of target genes that are differentially expressed in the roots and leaves at early and late infection stages in sugar beet by plant-derived microRNAs (miRNAs) include *Bevul.9G209500* (cytoplasm-related catalytic activity), *Bevul.2G095700* (potassium transporter) and *Bevul.9G160600* (zinc finger), which were up-regulated in the R line (vs. S). Viral-derived sncRNAs predominantly originated from RNA1 and RNA2 and targeted a subset of 69 sugar beet genes with overall expression that showed a strong negative correlation with higher sncRNA abundance. The results presented here for the first time demonstrate putative roles of sugar beet miRNAs in rhizomania resistance and BNYVV-derived sncRNAs and small peptides as potential pathogenicity factors.

## Data Availability

Sequence data for both mRNA and small non-coding RNA are deposited into the NCBI-SRA sequence repository under the project ID PRJNA1260505 and accession numbers in supplementary material (Table S7). All supplementary tables can be found via figshare: https://doi.org/10.6084/m9.figshare.30213841 .

## Introduction

Sugar beet (*Beta vulgaris* L.) is a key agronomic crop for sugar production in regions of the world with temperate climates. Among sources of sugar, 40% is produced from sugar beets with this proportion being much higher in temperate regions of the world such as Europe and the USA [1]. Among different biotic stresses resulting from viral and fungal pathogens that limit sugar beet productivity, rhizomania is one of the most globally impactful diseases that can cause up to 90% sucrose loss in case of high disease severity [2,3]. The predominant symptoms of rhizomania in sugar beet are numerous lateral rootlets on the main tap root accompanied by vascular tissue browning [4], yellow and narrow erect leaves and wilting of plants. The disease is caused by the pentapartite, positive-sense, single-stranded RNA virus, beet necrotic yellow vein virus (BNYVV), which is transmitted by *Polymyxa betae* [5], a soil-borne plasmodiophorid. While rhizomania is considered to have originated from Italy in the mid-20^th^ century, the disease can now be found in sugar beet growing regions around the world [3]. Since the first report of rhizomania in 1984 in the USA, the virus has widely spread across all major sugar beet growing regions in the country.

The genome of the BNYVV is composed of four or five RNA segments named RNA1 to 5 [6]. While the RNA5 fragment is usually absent in strains isolated from the USA, it is usually present in European and Asian strains [7]. Although the RNA5 fragment is not critical for BNYVV survival and pathogenicity of rhizomania disease, it has been shown to have a role in the disease development and severity [8]. The RNA fragments, RNA1 and RNA2, harbour genes that are known to function in viral replication, assembly, movement and suppression of plant antiviral RNA silencing during pathogenic interactions with host plant [8,9]. The RNA3 segment shows the most sequence variability among BNYVV isolates, and specific mutations within the ‘tetrad’ amino acid sequence of the p25 protein have been attributed to resistance breaking against *Rz1* [8,10]. The RNA4 plays important roles in BNYVV transmission by *P. betae*, enhancing disease symptoms and root-specific silencing suppression [11].

Rhizomania resistance in commercial cultivars is primarily dependent upon the use of two dominant resistant genes, *Rz1* and *Rz2* [3]. Additional resistant genes, namely, *Rz3*, *Rz4* and *Rz5*, have also been identified in sugar beets. Though questions have been raised if they can be considered as separate genes or are alleles of *Rz1* and *Rz2* as all *Rz* genes are localized in chromosome 3 in two separate resistant gene clusters [2]. Other approaches such as chemical control of rhizomania using soil fumigants have had some success but are not commercially viable due to cost, availability, etc. [12]. Management practices such as crop rotations and tillage are unable to control rhizomania at the commercial level [12,13]. To maintain sugar beet productivity, it is imperative to understand rhizomania infection and discover effective targets for future intervention as well as identifying new and novel sources of genetic resistance traits against rhizomania in sugar beets.

One underexplored aspect of plant–pathogen interaction related to rhizomania disease pathology and/or resistance in sugar beets is small non-coding RNAs (sncRNAs) that have been observed to accumulate in the plant host cells. These can either be derived from the plant host itself targeting BNYVV, or from BNYVV suppressing the plant host’s responses to the virus infection. Some of the most well-understood sncRNAs are microRNAs (miRNAs), which are typically 20–24 nt in length (highly conserved in plants) and can regulate complex biological processes through targeting mRNAs for degradation [14,15]. Host plant-derived miRNAs have been shown to regulate defence responses upon pathogen infection [16,18]. sncRNAs originating from viruses are also known for their roles in infection through interfering expression of genes (through RNA interference) associated with host defence response [19]. Studies have shown viral-derived sncRNAs targeting defence-related genes in plants such as Arabidopsis, tobacco and sugar beet and potentially acting as viral pathogenicity factors [17,20,22]. Virus-derived sncRNAs have also been implicated in host plant epigenetic modification through RNA-dependent DNA methylation [19]. Though some information is available on the production of sncRNA from RNA3 (ncRNA3) in BNYVV and its putative role as a pathogenicity factor [23], the roles of other sncRNAs produced by the BNYVV genome during natural infection of sugar beet plants, their putative roles in cross-kingdom RNAi and involvement in cellular processes leading to disease symptom severity are unknown. How sugar beet-derived miRNAs may contribute to rhizomania resistance by modulating the expression of key genes associated with relevant metabolic pathways besides targeting elements in the BNYVV genome is also unknown.

With the limited genetic resistance traits to rhizomania in current commercial sugar beet varieties – compounded with an increasing prevalence of resistance-breaking BNYVV strains that have been identified [10,24], there has become a renewed interest in sugar beet breeding programmes to identify new and novel sources of genetic resistance traits against rhizomania. Through chemical mutagenesis [ethyl methanesulphonate (EMS)] and phenotypic selection, we have previously reported on a potentially new source of rhizomania resistance, KEMS12 (PI 672570), which exhibited strong root and foliar resistance to rhizomania, observed as the lack of leaf yellow and lateral root nodules under field conditions [25]. KEMS12 was previously selected from 2,000 seeds from PI663873 (population C944), a random mating population that was treated with 1% EMS to induce mutations (https://npgsweb.ars-grin.gov/gringlobal/accessiondetail?id=1920336). The EMS-treated seeds were then evaluated for rhizomania resistance, and KEMS12 showed significant resistance against rhizomania compared to the untreated control (PI 663873) seedlings. The other EMS-treated line, KEMS09 (PI 672569), and another unrelated double-haploid KDH13 (PI 663862) line, which showed moderate and high susceptibility to rhizomania, respectively, were used for comparison to KEMS12. It should be mentioned that KEMS09 was initially released as a rhizomania-resistant line (https://npgsweb.ars-grin.gov/gringlobal/accessiondetail?id=1920335), but the resistance has been recently overcome [18]. Using natural infection in the field and comprehensive RNA (small RNA and mRNA) sequencing, we characterized sncRNAs across the three breeding lines corresponding to varying levels of genetic resistance to rhizomania. We further demonstrated the accumulation of specific BNYVV-derived sncRNAs and their putative roles during rhizomania pathogenic interactions with sugar beet and vice versa. Our results for the first time show miRNA-mediated regulation of genes known to be involved in metabolic pathways in sugar beet during resistant and susceptible interactions with BNYVV. The results also indicate the putative role of BNYVV-derived sncRNAs in cross-kingdom RNAi and modulating sugar beet gene expression.

## Methods

### Plant growth condition, viral infection of sugar beet plants and sample collection

**Fig. 1.**
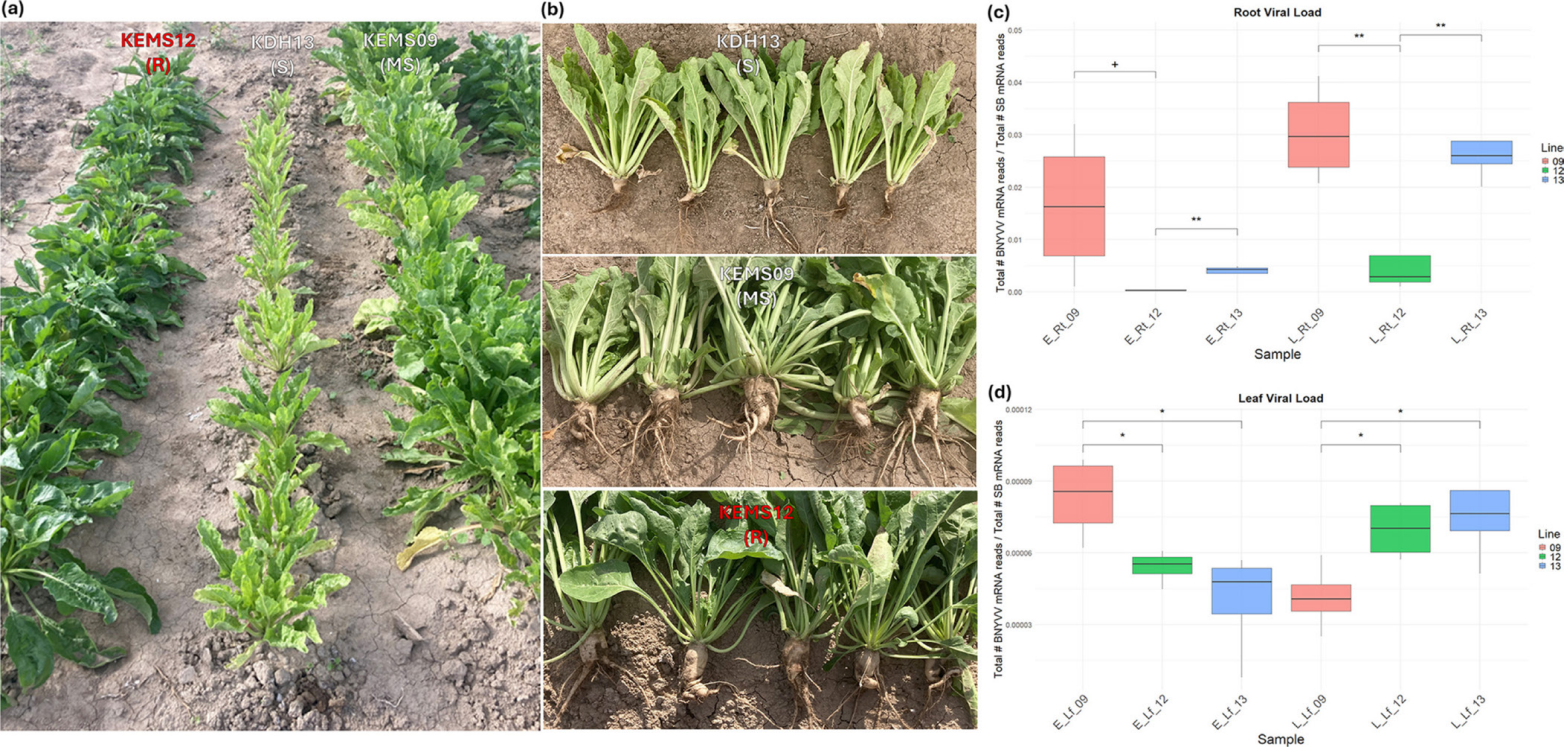
Disease phenotypes and viral load estimation in the rhizomania-resistant and -susceptible lines. (a) Plant stand in the rhizomania nursery at late infection stage. (b) Representative samples of uprooted plants at late infection stage. (c) Viral load in the roots. (d) Viral load in the leaves. Viral load was estimated as the ratio of total number of viral-derived transcripts/total number of sugar beet transcripts. Data are mean±SE of four biological replicates (eight individual plants/replicate). Rhizomania-resistant (KEMS12; 12), moderately susceptible (KEMS09; 09) and susceptible (KDH13; 13) sugar beet lines were used. ‘**’, *P* < 0.01; ‘*’, *P* < 0.05; and ‘+’, *P*<0.1 denote significant differences between lines for each treatment type. E_Rt, early root; L_Rt, late root; E_Lf, early leaf; L_Lf, late leaf.

A highly rhizomania-resistant (R) sugar beet EMS mutant breeding line KEMS12 (PI 672570), a moderately susceptible (MS) EMS mutant breeding line KEMS09 (PI 672569) and a highly susceptible (S) double-haploid line KDH13 (PI 663862) were evaluated in a sprinkler-irrigated sugar beet field [26] located in Kimberly, ID. Overall, KEMS09 did not show any major differences in viral load (vs. KDH13); the latter showed higher overall foliar and root stunting than KEMS09 (Fig. 1). We therefore designated KEMS09 as a moderately susceptible line and KDH13 as a highly susceptible line. The experiment was performed in a field that contained Portneuf silt loam soil and relied on natural infection of BNYVV for rhizomania development. The field was ploughed in the fall with a Terrano chisel plough. The field was fertilized (115 lb N and 140 lb P_2_O_5_/A), disked and roller harrowed in the spring, followed by transplanting of seedlings (2 weeks old and grown in greenhouse) during the first week of July. Each genotype was planted in four plots, each plot containing four rows (22-inch between-row spacing), and each row 24 ft long. Crop management was done following standard cultural practices in southern Idaho. Rhizomania foliar symptoms (yellow, upright and narrow leaves and stunted growth) were recorded during the fourth week of August, followed by hand digging of roots for sample collection. Roots were evaluated for rhizomania symptoms (numerous lateral rootlets, etc.) according to the method described in Strausbaugh *et al*. [26]. Leaf and root samples were collected in four replicates (each replicate comprised of tissues obtained from eight individual plants) at early (first week of August; 4-week post-transplanting; no visible disease symptom) and late (fourth week of August; 8-week post-transplanting; major disease symptoms) infection stages, flash frozen in liquid N, and stored in −80 °C freezer until further processing. As sugar beet fields in southern Idaho contain some level of BNYVV inoculum in the soil, we chose two different stages, namely, early and late, to analyse plant samples for this work. The samples were pulverized at −80 °C with a Geno/Grinder 2010 (SPEX SamplePrep; Metuchen, NJ, USA). The sugar beet lines used in this study are highly homozygous and have been evaluated for rhizomania resistance/susceptibility during multiple growing seasons at the USDA-ARS, Kimberly, ID, rhizomania disease nursery [25,27, 28].

### Extraction of total RNA, sRNA and mRNA library preparations and sequencing

Total RNA was extracted using the ‘Plant/Fungi Total RNA Purification Kit’ (Norgen Biotek Corp, ON, Canada) according to the manufacturer’s protocol. The quality and quantity of RNA were determined using Bioanalyzer 2100 (Agilent Technologies, Santa Clara, CA, USA). RNA samples showing a RNA integrity number >7.0 were subsequently used to construct small RNA sequencing (sRNAseq) and mRNA sequencing (mRNAseq) libraries. Approximately 1 ug of total RNA was used to prepare sRNAseq and mRNAseq libraries. Illumina HiSeq 2500 sequencing platform and 50 bp single-end sequencing approach were used for sRNA sequencing at LC Sciences (Houston, TX, USA) according to the vendor’s protocol [22]. rRNA depletion was performed for mRNAseq libraries using the Ribo-Zero™ rRNA Removal Kit (Illumina, San Diego, CA, USA). Poly(A) mRNA was purified using oligo-(dT) magnetic beads. Poly(A) RNA was fragmented using a divalent cation buffer at elevated temperature, which was followed by reverse-transcription to produce cDNA that was used to produce U-labelled second strand DNA. Final libraries were made following end repair, 3′ adenylation, adapter ligation and PCR. For quantification and quality control of mRNAseq libraries, Bioanalyzer 2100 was used. The libraries were sequenced on Illumina’s NovaSeq 6000 sequencing platform using paired-end (150 bp) sequencing approach.

### Read mapping and transcriptome assembly

The small RNA-seq and mRNA-Seq raw reads were processed using in-house (LC Sciences) Perl scripts and Cutadapt (1.10) [29]. Removal of low-quality reads and adapter sequences was followed by FastQC (0.10.1) (http://www.bioinformatics.babraham.ac.uk/projects/fastqc/) for sequence quality evaluation. The reads were then mapped to the EL10.2 (https://phytozome-next.jgi.doe.gov/) sugar beet (*B. vulgaris* subsp. *vulgaris*) reference genome using Hisat (2.0) [30]. The mapped reads resulting from each sample were assembled using StringTie (1.3.4) [31].

### Bioinformatic analysis of miRNAs

Raw reads originating from sRNAseq were processed by removing adapter dimers, low quality reads, repeats, rRNA, tRNA, small nuclear RNA and small nucleolar RNA sequences using an in-house programme, ACGT101-miR (LC Sciences). Unique sequences ranging between 18 and 25 nt in length were mapped to specific miRNA precursors in the miRBase 22.0 database (https://www.mirbase.org/) to identify known and novel miRNAs. Parameters including nucleotide (nt) variations at 3′ and 5′ ends and one mismatch inside the sequence were taken into consideration during alignment. miRNAs that mapped to the specific species of mature miRNAs in the hairpin arms were designated as known miRNAs. The miRNA sequences that mapped to the other arm of known miRNA species precursor hairpin sequences opposite to the annotated mature miRNA-containing arm were designated as novel 5p- or 3p region-derived miRNAs. The remaining small RNA sequences were mapped to other selected miRNA species precursors (using blast search) in the miRBase 22.0, and the mapped pre-miRNAs were blast analysed against the sugar beet genome to determine their locations in the genome. The unmapped small RNA sequences were blast analysed against the sugar beet genome. Hairpin RNA structures in the sequences were predicted from the flanking 120 nt sequences using RNAfold (http://rna.tbi.univie.ac.at/cgi-bin/RNAWebSuite/RNAfold.cgi). Copy number rectification of miRNAs among different samples [32] was performed using a global normalization method that helped to overcome any biasedness originating from sequencing discrepancy on miRNA expression. Differentially expressed (DE) miRNAs were those that showed *P*<0.05 and fold change ≥1.5. miRNA target gene prediction in sugar beet was performed using the target prediction algorithm PsRobot 1.2 [33]. Further miRNA target validation of sugar beet genes was performed by using mRNAseq data and comparing the expression of target genes to the abundance of specific miRNAs that target those genes.

### Differential expression of mRNAs and bioinformatic analysis

Transcriptome data obtained from 48 different samples were merged to reconstruct comprehensive transcriptome data using Perl scripts. StringTie (1.3.4) [31] and edgeR (3.42) [34] were used for transcript quantification and expressed as fragments per kilobase of transcript per million mapped reads (FPKM). The EdgeR package was used for differential expression analysis. A *P*-value of <0.05 and | log2 (fold change) | ≥ 2 or ≤−2 were used to identify DE genes. Annotation of transcripts was performed using blastx against the NCBI database. For Gene Ontology (GO) analysis, transcripts were aligned via blast against the GO database to calculate the gene numbers for each term. Pathway enrichment was performed using the Kyoto Encyclopedia of Genes and Genomes (KEGG) [35]. The R line (KEMS12) was compared to the susceptible lines (KDH13 and KEMS09) both at early and late infection stages.

### Characterization of BNYVV sncRNAs

The sncRNA from leaf and root samples that originated from the BNYVV genome was identified. Total sncRNA sequences were aligned to the BNYVV genome (NCBI GCF_000854885.1) using ‘bwa aln’ [36], requiring 100% read identity using ‘-n 0’. The lengths of these reads were compared to the length of the total sncRNA. To identify possible targets in the sugar beet host, the reads attributed to the BNYVV virus were aligned to the EL10.2 sugar beet genome (https://phytozome-next.jgi.doe.gov/). This alignment was done using blastn [37] to allow for more sensitive alignment given the short nature of the data: ‘blastn -gapopen 2 -gapextend 1 -word_size 7 -evalue 0.05’. The mapped locations of these sncRNAs were compared to gene annotations, and correlations between the abundance of sncRNAs and their effect on target gene expression were performed using R ‘lm(FPKM ~sncRNA count)’ function. GO term enrichment analysis was performed using the R package ‘topGO’, comparing all genes with BNYVV aligned sncRNA to all other genes [38].

### Peptide extraction and processing

Approximately 100 mg of finely ground sugar beet root tissues (from 32 individuals of each KEMS12 resistant and KDH13 susceptible lines) were extracted in 1,000 µl of 75% methanol in vials containing steel beads, ground at 70 HZ for 3 min and followed by ultrasonication in an ice-water bath for 20 min. The grinding and ultrasonication steps were repeated twice. This was followed by incubation at −40 °C for 1 h, centrifugation at 12,000 r.p.m. for 10 min at 4 °C, transferring a 900 µl volume of the supernatant to new tubes, and vacuum freeze-dried until further use. Later, a 200 µl volume of wash buffer was used to dissolve the sample. Activation of the desalting column was performed by using 100 µl of methanol to the desalting column and centrifugation at 700 ***g*** for 1 min. This was followed by desalting column impurity removal by adding 100 µl of conditioning buffer to the desalting column and centrifugation at 700 ***g*** for 1 min. Desalting column equilibration was done by adding 100 µl wash buffer to the desalting column and centrifugation at 700 ***g*** for 1 min. A 100 µl volume of sample was loaded into the desalting column and centrifuged at 700 ***g*** for 1 min. This was followed by column washing with wash buffer and eluting peptides in a final volume of 100 µl. The samples were concentrated using a vacuum refrigerated centrifugal concentrator and re-dissolved in mobile phase A, and equal amounts of peptides were used for MS analysis.

### MS analysis

For each sample, ~200 ng of total peptides was separated and analysed using a nano-UPLC (Evosep One; Odense, Denmark) coupled to a timsTOF Pro2 instrument (Bruker, Germany) with a nano-electrospray ion source. Separation was performed using a reversed-phase column (PePSep C18 column, 1.9 µm, 150 µm×15 cm; Bruker). Mobile phases were H_2_O with 0.1% formic acid (phase A) and acetonitrile with 0.1% formic acid (phase B). The mass spectrometer adopts data-dependent acquisition-parallel accumulation-serial fragmentation (DDA PaSEF) mode for DDA data acquisition, and the scanning range was from 100 to 1,700 m/z for MS1. During PASEF MS/MS scanning, the impact energy increased linearly with ion mobility, from 20 eV (1 /K0=0.6 Vs cm^2^) to 59 eV (1 /K0=1.6 Vs cm^2^).

### Peptidome data analysis

Raw MS files were processed using the SpectroMine software (4.2.230428.52329) with the built-in Pulsar search engine according to the standard methods followed by the vendor (Lifeasible; Shirley, NY, USA). MS spectra lists were searched against their species-level UniProt FASTA databases (uniprotkb_taxonomy_id_161934_2025_01 20.fasta), carbamidomethyl (C) as a fixed modification, oxidation (M) and acetyl (protein N-term) as variable modifications. No-enzyme (unspecific) setting was used. The false discovery rate was set to 0.01 for both peptide-spectrum match and peptide levels. Peptide identification was performed with an initial precursor mass deviation of up to 20 p.p.m. and a fragment mass deviation of 20 p.p.m. All other parameters were kept as default.

### Identification of small peptides putatively derived from BNYVV

Small peptides putatively originating from the virus were evaluated by analysing small ORFs (sORFs) and searching for peptide sequences in the BNYVV genome. The NCBI ORF finder tool (https://www.ncbi.nlm.nih.gov/orffinder/) with minimum ORF length of 30 nt, including ‘ATG’ and alternative initiation codons, was used. We then used blastn [37] to search for these proteins from these ORFs and blastx to search the BNYVV genome for evidence of the sequenced small peptides.

## Results

### Differential expression of miRNAs in the roots and leaves of R vs. S lines at early and late infection stages

**Fig. 2.**
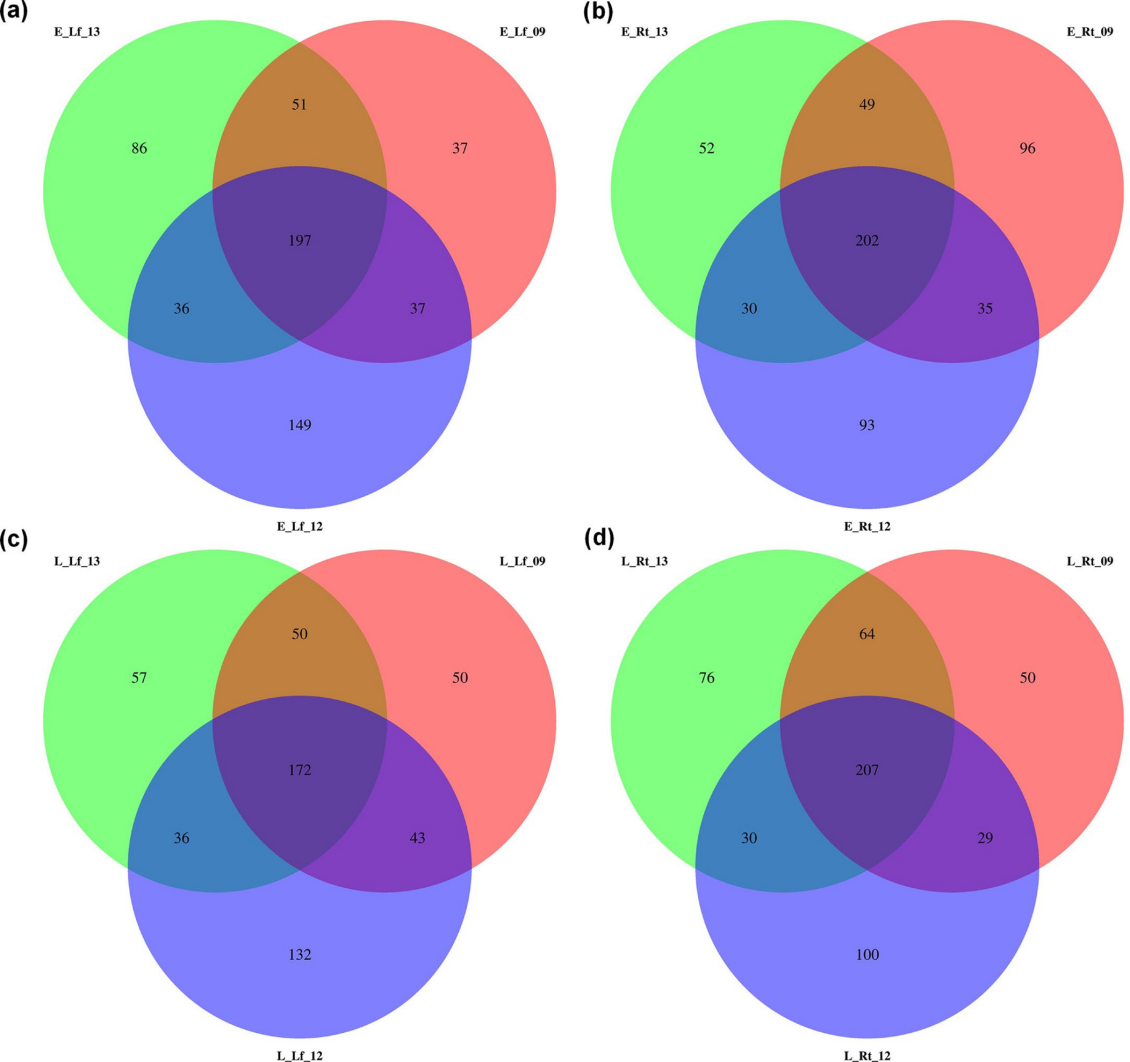
Venn diagrams of DE miRNAs at different stages of rhizomania infection. (a) Early root (E_Rt). (b) Early leaf (E_Lf). (c) Late root (L_Rt). (d) Late leaf (L_Lf). Rhizomania-resistant (KEMS12; 12), moderately susceptible (KEMS09; 09) and susceptible (KDH13; 13) sugar beet lines were used. Data are mean±SE of four biological replicates (eight individual plants/replicate).

**Fig. 3.**
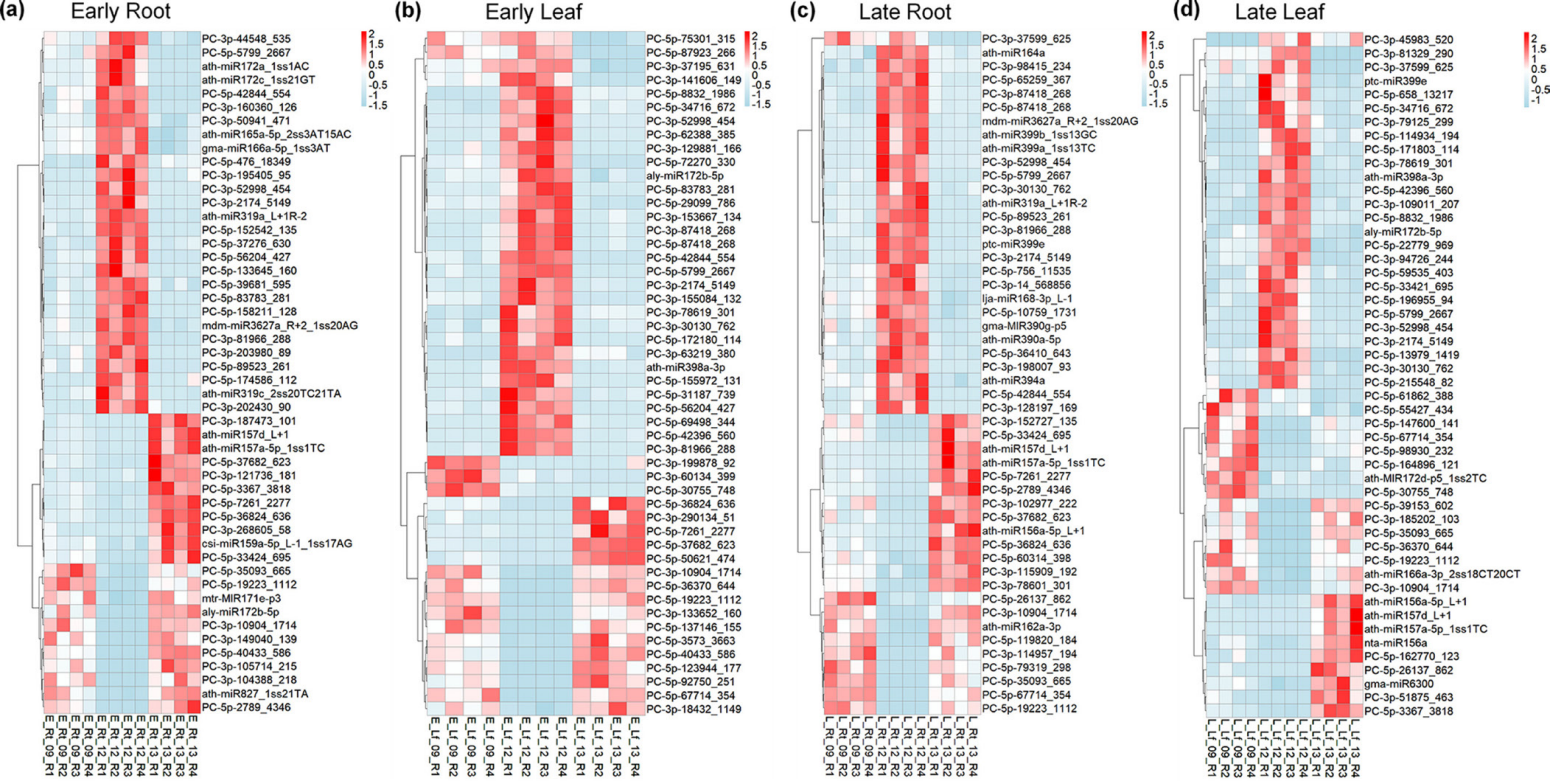
Heatmaps of the top 50 DE miRNAs (*P* < 0.0005) at different stages of rhizomania infection. (a) early root (E_Rt), (b) early leaf (E_Lf), (c) late root (L_Rt) and (d) late leaf (L_Lf) in the rhizomania-resistant (KEMS12; 12), moderately susceptible (KEMS09; 09) and susceptible (KDH13; 13) sugar beet lines. Data are from four biological replicates (eight individual plants/replicate).

DE miRNAs were analysed in the rhizomania-resistant and -susceptible lines at early (no visible disease symptom) and late (major disease symptoms) infection stages to understand the regulatory role of sugar beet miRNAs in resistance and susceptibility. The R line showed higher root resistance and resistance to foliar symptoms against BNYVV vs. S lines phenotypically (Fig. 1a, b). Viral load was lower (*P*<0.05) in the roots of R line vs. S lines at the late infection stage (Fig. 1c). In the leaves, the number of viral reads observed was extremely low, as expected due to the root infection nature of the disease and showed no consistent pattern (Fig. 1d). The samples showed an average sequencing depth of ~11 million raw reads/sample (Table S1, available in the online Supplementary Material). The number of DE miRNAs unique to the R lines was generally higher both in the roots and leaves at early and late infection stages (Fig. 2). Heatmaps of the top 50 DE miRNAs (*P*<0.0005) at early and late infection stages in the roots and leaves of R and S lines are shown in Fig. 3. The complete list of DE miRNAs (*P*<0.05) is presented in Table S2. At early infection stage, examples of miRNAs that showed moderately high expression only in the roots of R line (vs. no expression in the S lines) included PC-5p-83783_281, PC-3p-81966_288, PC-5p-89523_261, etc. (Fig. 3a), whereas miRNAs that were up-regulated (~4- to 9-fold) in the roots of R line (vs. S lines) at this stage included ath-miR319a_L+1 R-2, PC-5p-39681_595 etc. Conversely, miRNAs that were down-regulated (~10- to 12-fold) in the roots of R line (vs. S lines) at this stage included ath-miR157a-5p_1ss1TC, ath-miR157d_L+1, etc. and no expression in the R line such as PC-3p-187473_101. miRNAs that were highly up-regulated in the early infection stage leaves of R line (vs. S lines) included PC-5p-155972_131 (no to minimal expression in S lines), ath-miR398a-3p, PC-5p-83783_281 (no to minimal expression in S lines), etc. (Fig. 3b), whereas miRNAs that were down-regulated in the leaves of the R line (vs. S lines) at this stage included PC-3p-10904_1714 (77 to 81-fold), PC-5p-36370_644 (no expression in R line), etc.

At the late infection stage, miRNAs that were highly up-regulated in the R line (vs. S lines) in the roots included PC-3p-2174_5149 (~10- to 16-fold), PC-5p-89523_261 (no to minimal expression in S lines), PC-3p-81966_288 (no to minimal expression in S lines), PC-3p-128197_169 (no to minimal expression in S lines), etc. (Fig. 3c). Conversely, the expression of miRNAs down-regulated in the roots of R line (vs. S lines) at this stage included ath-miR162a-3p (~2.5-fold), PC-3p-10904_1714 (no expression in R line), etc. In the leaves, examples of highly up-regulated miRNAs in the R line (vs. S lines) included PC-5p-196955_94 (no to minimal expression in S lines), PC-3p-30130_762 (~2- to 3-fold), PC-5p-171803_114 (no to minimal expression in S lines), etc. Examples of miRNAs down-regulated (no expression) in the leaves of R line (vs. S lines) at this stage included PC-5p-39153_602, PC-3p-185202_103, etc. (Fig. 3d).

### GO and KEGG targeted by DE miRNAs

**Fig. 4.**
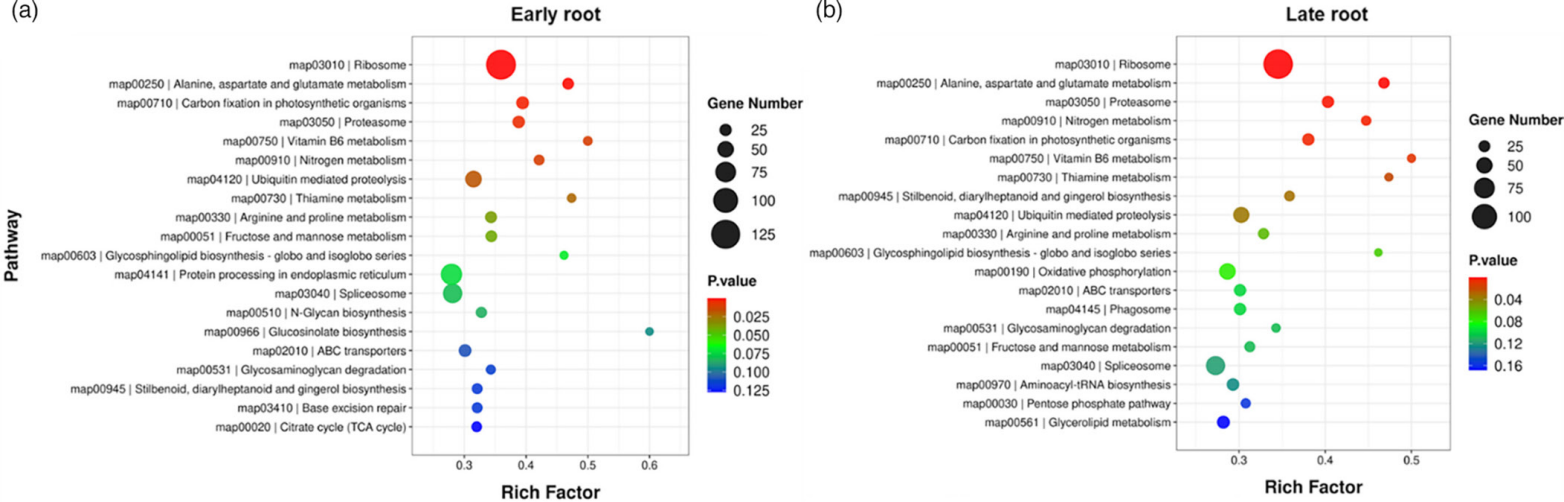
KEGG enrichment of sugar beet target genes of DE sugar beet miRNAs at different stages of rhizomania infection. (a) Early root. (b) Late root. Data are mean±SE of four biological replicates (eight individual plants/replicate). DE miRNAs target sugar beet genes that alter their expressions.

GO analysis revealed DE miRNAs (considering roots and leaves at early and late infection stages) targeted sugar beet genes primarily associated with cellular processes such as cytosolic large and small ribosomal subunit, structural constituent of ribosome and lignin biosynthesis to name a few (Fig. S1). Pathway enrichment analysis of target genes by DE miRNAs represented pathways predominantly associated with ribosome, alanine, aspartate, glutamate metabolism, photosynthetic carbon fixation and proteasome in the roots and leaves at early and late infection stages (Figs 4 and S2).

For mRNAseq, we obtained >42 million raw reads/sample and >38 million valid reads/sample (Table S3). In different situations, we observed the same sugar beet gene putatively targeted by multiple miRNAs and vice versa and reduced the expression of target genes in the R and S lines. Examples of some strong negative correlations between higher and lower abundance of miRNAs in the roots and leaves at early and late infection stages and altered gene expressions are shown in Table 1. An elaborate list of miRNA target sugar beet genes and their effects on expression is presented in Table S4. Down-regulation of ath-miR157a-5p_1ss1TC and ath-miR157d_L+1 in the roots of KEMS12 and KEMS09 lines (vs. S) at early infection stage increased the expression of the same target gene, *Bevul.9G209500* (cytoplasm related catalytic activity), by ~2-fold. Conversely, up-regulation of ath-miR319a_L+1 R-2 in the R line reduced the expression of its target, *Bevul.9G175500* (serine (ser)/threonine (thr)-protein kinase), by >7-fold. At the late infection stage, down-regulation of ath-miR162a-3p in the roots of R line increased the expression (vs. S) of its targets *Bevul.2G095700* (~3-fold; potassium transporter) and *Bevul.9G160600* (7-fold; zinc finger). In symptomatic leaves at this stage, down-regulation of PC-3p-81329_290 and PC-5p-42396_560 in the R line (vs. S) reduced the expression of the same target gene, *Bevul.2G122300* (MIP aquaporin), by >4-fold.

**Table 1.** DE sugar beet miRNAs that targeted sugar beet genes and showed changes in target gene expression Rhizomania-resistant (KEMS12; 12), moderately susceptible (KEMS09; 09) and susceptible (KDH13; 13) sugar beet lines were used. Data are mean±SE of four biological replicates (eight individual plants/replicate). E, early; L, late; Rt, root; Lf, leaf.

Sugar beet gene	Description	Tissue	Time	miRNA	Interaction *P*-value	FPKM- 09	FPKM-12	FPKM-13	miRNA FPKM-09	miRNA FPKM-12	miRNA FPKM-13
Bevu1.2G013300	ADP binding	Lf	E	PC-5p-30755_748	3.61E-07	0.27	1.00	1.00	48.75	5.25	1.00
Bevu1.5G113900	Cysteine and methionine metabolism	Lf	E	PC-5p-36370_644	1.21E-07	740.99	1,884.90	345.10	44.50	1.00	50.75
Bevu1.2G139500	Cytoplasm-related catalytic activity	Rt	E	PC-3p-187473_101	3.49E-06	76.17	74.19	34.47	3.75	1.00	41.50
Bevu1.9G175500	ser/thr protein-kinase	Rt	E	ath-miR319a_L+1 R-2	2.14E-09	1.00	0.14	1.00	131.00	587.50	58.75
Bevu1.9G209500	Cytoplasm-related catalytic activity	Rt	E	PC-5p-36824_636	9.62E-09	20.58	23.21	10.29	15.00	1.00	66.75
Bevu1.9G209500	Cytoplasm-related catalytic activity	Rt	E	ath-miR157a-5p_1ss1TC	1.20E-07	20.58	23.21	10.29	51.50	22.25	273.75
Bevu1.9G209500	Cytoplasm-related catalytic activity	Rt	E	ath-miR157d_L+1	1.20E-07	20.58	23.21	10.29	51.50	22.25	273.75
Bevu1.2G122300	MIP aquaporin	Lf	L	PC-3p-81329_290	1.52E-07	11.67	4.33	19.46	7.75	11.00	1.00
Bevu1.2G122300	MIP aquaporin	Lf	L	PC-5p-42396_560	5.24E-06	11.67	4.33	19.46	5.00	45.75	4.25
Bevu1.2G095700	Potassium transporter	Rt	L	ath-miR162a-3p	4.52E-06	9.31	19.05	6.36	2,077.75	835.00	2,086.5
Bevu1.9G160600	Zinc finger	Rt	L	ath-miR162a-3p	6.92E-06	5.03	21.12	3.10	2,077.75	835.00	2,086.5

### BNYVV-derived sncRNAs and their putative target gene sugar beet

**Fig. 5.**
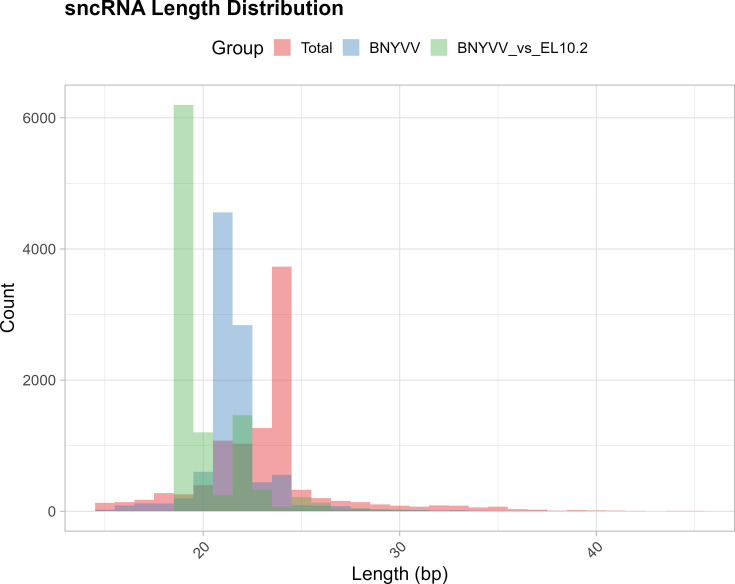
Length distribution of sncRNAs across sugar beet root and leaf tissues at early and late infection stages. The sncRNAs were classified into three categories: all sncRNA (red), those putatively derived from BNYVV (blue) and BNYVV sncRNA alignment lengths to the EL10.2 sugar beet genome (green). Each category was sampled to 10,000 reads for comparison.

A subset of sncRNAs that originated from the BNYVV genome was characterized. The proportion of the sncRNAs attributable to the BNYVV genome ranged between 0 and 1.3%. The lengths of these sncRNAs primarily fell within 21–22 nt compared to a larger distribution of length (~15–40 nt) of the total sncRNAs (Fig. 5). These BNYVV sncRNAs were then aligned to the sugar beet genome to evaluate potential host plant targets of the viral-derived sncRNAs. Approximately 1% of the BNYVV sncRNAs had putative targets in the sugar beet genome, amounting to anywhere from tens to a couple of thousand total sncRNA reads. The aligned portion of the BNYVV sncRNAs that showed sequence complementarity to the sugar beet genome was enriched for 19–20 bp in length. The majority of sncRNAs derived from BNYVV fell within RNA1 and RNA2 of the five viral genome segments (Fig. S3). No sncRNAs were detected from the RNA5 segment of BNYVV.

**Fig. 6.**
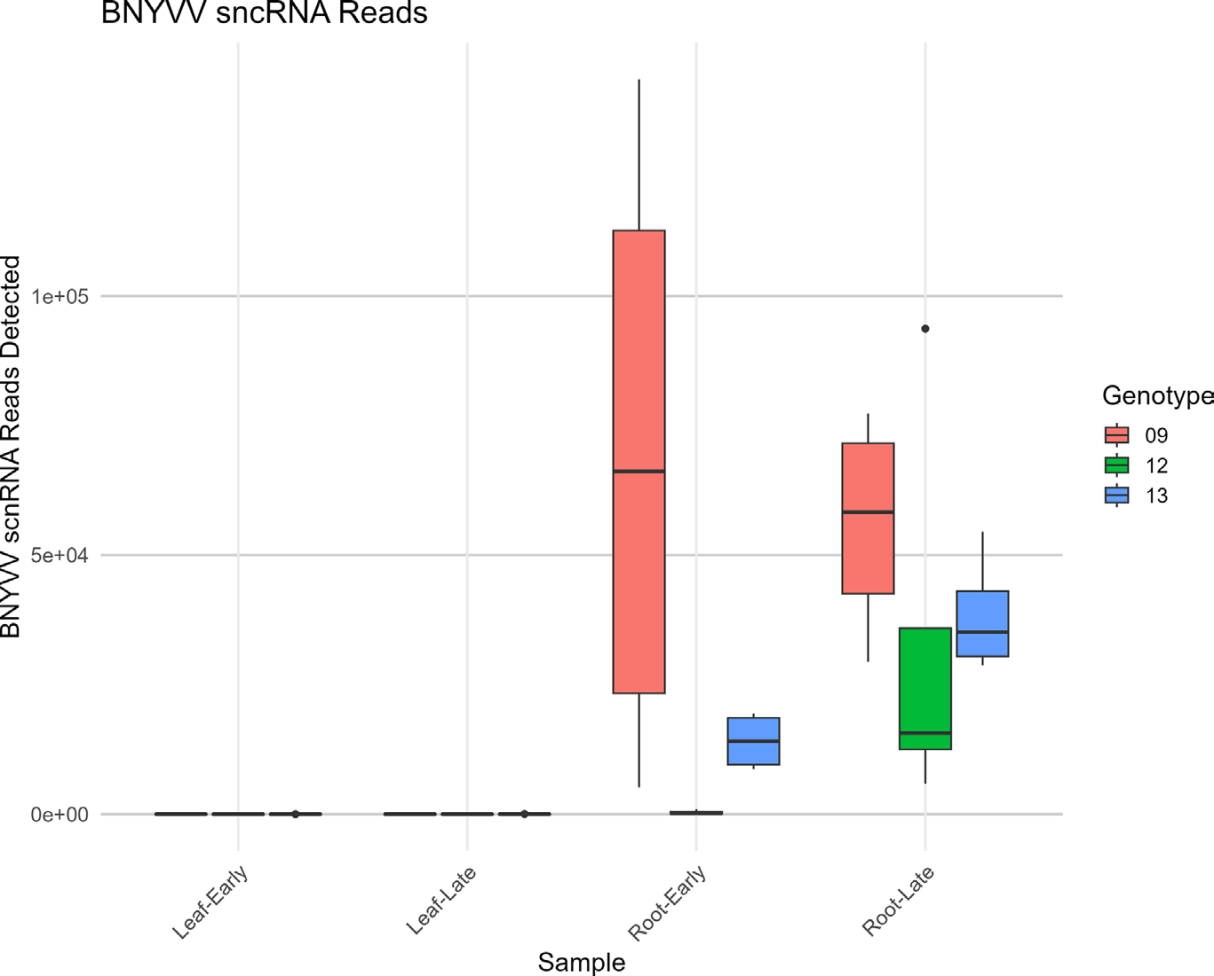
Abundance of BNYVV-derived sncRNAs in the beet roots and leaves of rhizomania-resistant (KEMS12; 12), moderately susceptible (KEMS09; 09) and susceptible (KDH13; 13) sugar beet lines at early and late infection stages. Data are mean±SE of four biological replicates (eight individual plants/replicate).

The abundance of BNYVV sncRNAs was compared among all sugar beet samples (Fig. 6). Very little BNYVV scnRNA reads were observed in the leaves compared to the roots, regardless of infection stages and genotypes. While there was a high variation among samples, the R line (KEMS12) consistently showed lower BNYVV sncRNA abundance in the roots at early and late infection stages.

To dissect possible targets of the BNYVV sncRNAs in the sugar beet genome, we compared the aligned sncRNA read locations to sugar beet genome as well as changes in gene expression across the three lines. The aligned BNYVV sncRNAs overlapped with a total of 69 genes (Tables 2 and S5, Fig. S4). These genes are spread across all sugar beet chromosomes, with an enrichment of sncRNAs mapping to chromosome 3 (Fig. S5). Many of the sncRNAs mapped to multiple locations in the sugar beet genome with high fidelity, indicating the potential for multi-target activity. As an example, the sncRNA with near identical sequence (CAAAACCACCACCACCACC) aligned to four separate locations along chromosome 3 (Fig. S5). We performed GO term enrichment for biological processes influenced by the 69 genes with aligned BNYVV sncRNA (Table 3). While many of the biological processes overlap, notable pathways among them are the ethylene and jasmonic acid mediated signalling pathway.

**Table 2.** The putatively viral-derived sncRNAs with highest overall expression across samples and their sequence homology to sugar beet genes The sncRNA sequence is given with nucleotides in brackets showing presence/absence variation among reads. Asterisks indicate mismatched nucleotides between reads and the sugar beet reference genome. The percent identity within aligned regions is displayed, which only considers the core motif aligned to the sugar beet genome.

Sequence	RNA segment	Total sncRNA read count	Gene	Function	Identity (%)
[G*U*]CAAAACCACCACCACCACC[A*G*A*]	2	5788	Bevul.3G005600, Bevul.5G005900	Glycine-rich cell wall structural protein, lipid phosphate phosphatase beta	100
[GC*GA]AAAAUGGUCAUGUCGGA[AGC*A]	1	821	Bevul.2G137100	Peroxisome biogenesis factor	100
AAAACUGCUGGCA*CUAAUGAGA[A*U*]	2	685	Bevul.2G003200	B3 domain-containing protein	95
[A*]U*GAUUGUAGCCUGUGGGUUG[U*U*A*U*]	3	407	Bevul.7G091100	Alkylated DNA repair protein alkB homologue	100
UUGUUGUUGGUGUUU*GUGUUGU	2	121	Bevul.6G178500	na	95
CCCUACAAGGACUGGUACAUU*	3	112	Bevul.4G184200	Belongs to the peptidase A1 family	100
[U]UG*GACAAUGGUGCUGAUGAGU	1	95	Bevul.6G112200	na	100
[U*]GAAAGUGUUGAGG*GUGUGGAAC[G*]	2	95	Bevul.6G240300	LRR receptor-like serine threonine-protein kinase	95
UCAACGAGUUG*GUGAUGAGCUU	1	84	Bevul.1G057900	Anaphase-promoting complex subunit	95

NA, genes with an unknown function.

**Table 3.** GO term enrichment for sugar beet genes with sncRNA putatively derived from BNYVV mapped against them GO terms are determined as the predicted biological process. *P*-value was determined by classic Fisher test comparing the number of total, significant and expected genes containing the GO term.

GO.ID	Term	Annotated	Significant	Expected	*P*-value
GO:0006900	Vesicle budding from membrane	15	2	0.05	0.0012
GO:0010193	Response to ozone	23	2	0.08	0.0029
GO:0000122	Negative regulation of transcription	35	2	0.12	0.0066
GO:0010119	Regulation of stomatal movement	60	2	0.21	0.0186
GO:0009873	Ethylene-activated signalling pathway	63	2	0.22	0.0204
GO:0009867	Jasmonic acid-mediated signalling pathway	66	2	0.23	0.0223
GO:0043043	Peptide biosynthetic process	307	4	1.08	0.0259
GO:0009260	Ribonucleotide biosynthetic process	70	2	0.25	0.027
GO:0045737	Positive regulation of cyclin-dependent kinase activity	10	1	0.04	0.0346
GO:0070071	Proton-transporting two-sector ATPase	10	1	0.04	0.0346

**Fig. 7.**
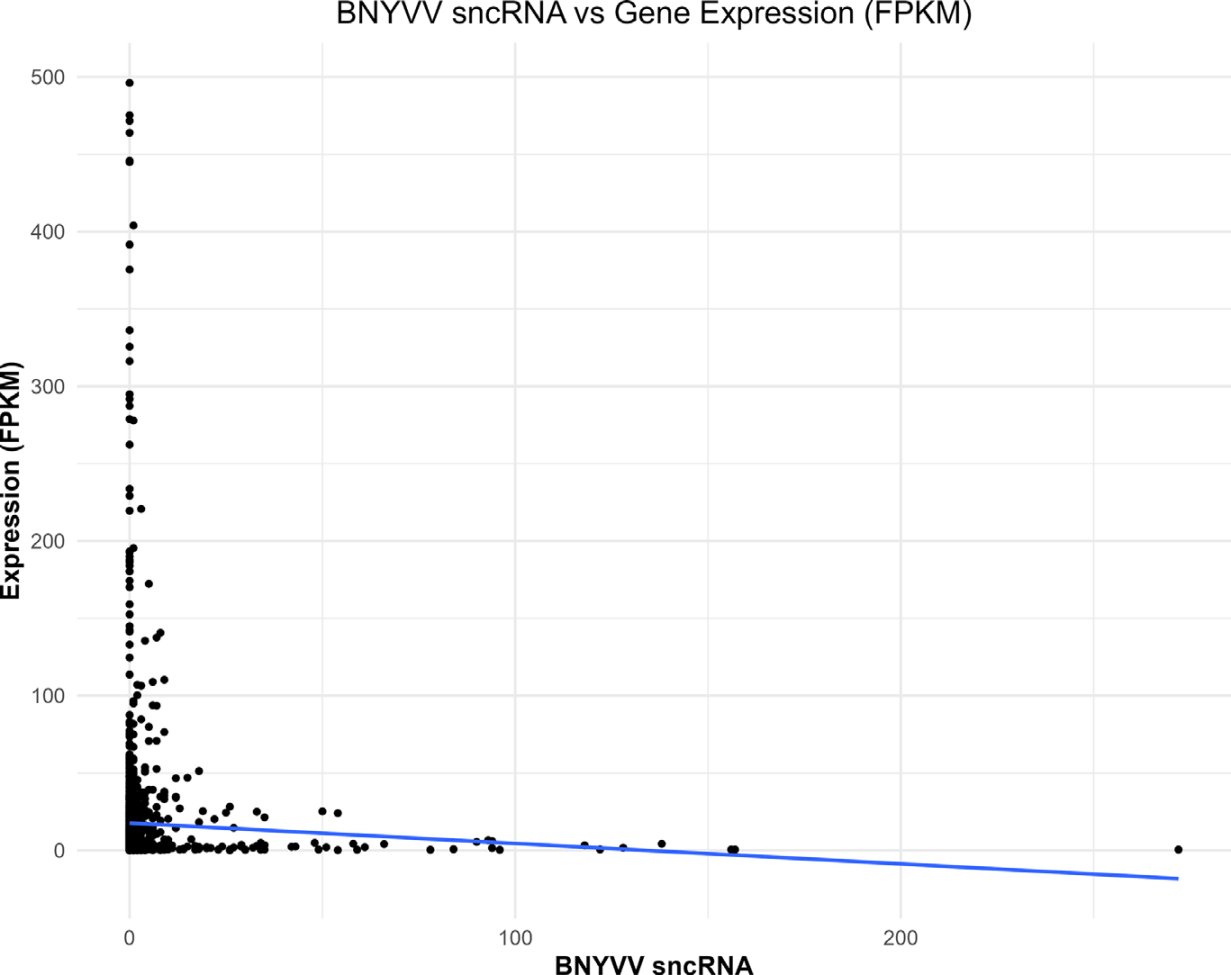
BNYVV sncRNA abundance and sugar beet mRNA expression. Scatterplot of sugar beet gene expression and count of sncRNAs.

A linear correlation analysis of the abundance of BNYVV sncRNA mapping to specific sugar beet genes with their respective expression of those target genes was performed. Gene expressions were measured across the same genotypes, infection stages (early and late) and replicates as the BNYVV sncRNA data. Considering each of the 69 genes, there were no significant effects on gene silencing where high BNYVV sncRNA mapping to the gene implied lower measured mRNA abundance (Fig. S6) aside from one single gene with little meaningful variation (Fig. S7). However, when looking at all 69 genes together, we found that a higher abundance of BNYVV sncRNAs was significantly associated with lower expression of those gene clusters (*P*=0.0024, Fig. 7).

### Sugar beet miRNAs target BNYVV genome

We also investigated if sugar beet miRNAs have putative targets in the BYVV genome. A list of DE miRNAs that putatively targeted BNYVV genes is shown in Table 4. In general, the majority of these highly significant sugar beet miRNAs primarily targeted BNYVV RNA-dependent RNA polymerase, replicase and 42 kDa transport protein located in RNA1 and RNA2 segments. Some examples include up-regulation (>2-fold in the R line vs. S) of aly-miR172b-5p targeting RNA-dependent RNA polymerase in the leaves at early and late infection stages, up-regulation (>4-fold in the R line vs. S) of ath-miR172c_1ss21GT targeting 42 kDa transport protein in the roots at early and late infection stages, etc.

**Table 4.** DE sugar beet miRNAs and their putative targets in the BNYVV genome (NCBI GCF_000854885.1) Rhizomania-resistant (KEMS12; 12), moderately susceptible (KEMS09; 09) and susceptible (KDH13; 13) sugar beet lines were used.

Sample tissue	Infection stage	Sugar beet miRNA	miRNA sequence	*P*-value (differential expression)	miRNA abundance (FPKM) line 09	Line 12	Line 13	Alignment identity	Alignment match	RNA segment	Start	End
Root	Late	PC-3p-30062_764	GTGAAGTGGACTCTCTCTCCTTA	0.000326	16	12	31	100	12	1	4,356	4,345
Leaf	Early	PC-5p-137146_155	AGATATATTCCGTGTACCTCAACC	4.38E-05	17.75	0	18	100	12	1	3,682	3,671
Leaf	Early	aly-miR172b-5p	ACAGCACCATCAAGATTACCA	0.000125	109.75	221.25	88.5	100	12	1	4,258	4,247
Root	Early	aly-miR172b-5p	GCAGCACCATCAAGATTACCA	4.69E-05	6.25	0	7.75	100	12	1	4,258	4,247
Leaf	Late	aly-miR172b-5p	GCAGCACCATCAAGATTACCA	7.79E-05	136.5	303	114.25	100	12	1	4,258	4,247
Root	Early	PC-5p-33424_695	TTTTTGAAACATAGCCTGGAT	7.61E-05	25.5	0	50	100	12	1	486	475
Root	Late	PC-5p-33424_695	TTTTTGAAACATAGCCTGGAT	0.000185	49.25	0	93.25	100	12	1	486	475
Root	Early	ath-miR172a_1ss1AC	CGAATCTTGATGATGTCGCAT	8.03E-05	264	487.25	99.25	100	13	2	3,109	3,121
Root	Late	ath-miR172c_1ss21GT	CGAATCTTGATGATGTCGCAT	0.000117	229	424.75	84.75	100	13	2	3,109	3,121
Root	Late	ath-miR172c_1ss21GT	AGAATCTTGATGATGTCGCAT	0.000453	579.75	686	152.25	100	13	2	3,109	3,121
Root	Late	ath-miR162a-3p	TCGATAAACCTCTGCATCCAG	2.73E-06	2,149.5	849	2,286.75	85.714	21	5	75	55

To consider another possible dimension of pathogen–host interaction, we obtained small peptide data from a subset of our samples. These small peptides ranged between 7 and 30 aa in length. We searched the BNYVV genome for evidence of putative viral-derived small peptides. The peptides identified in this study did not correspond to any annotated sORFs; however, there were three peptides that corresponded to small portions of 75 and 14 kDa proteins derived from the main ORFs located in RNA segment 2 (Table 5, Fig. S4). These three peptides varied in whether they were observable in the resistant (KEMS12) and susceptible (KDH13) lines.

**Table 5.** Small peptides with matching sequences among proteins derived from BNYVV Their presence and/or absence in the root samples obtained at the late infection stage from the susceptible (KDH13) and resistant (KEMS12) lines are designated by positive (+) and negative (−) symbols.

Peptide sequence	Peptide length	KDH13	KEM12	BNYVV gene	Gene description	RNA segment	Start	End
IADEEQRTL	9	+	–	BNYVVs2gp1	75 kDa protein	2	1,576	1,602
HHVIVDPEV	9	–	+	BNYVVs2gp6	14 kDa protein	2	4,394	4,420
RLESSRVEATHAVA	14	–	+	BNYVVs2gp1	75 kDa protein	2	1,609	1,650

## Results summary

**Fig. 8.**
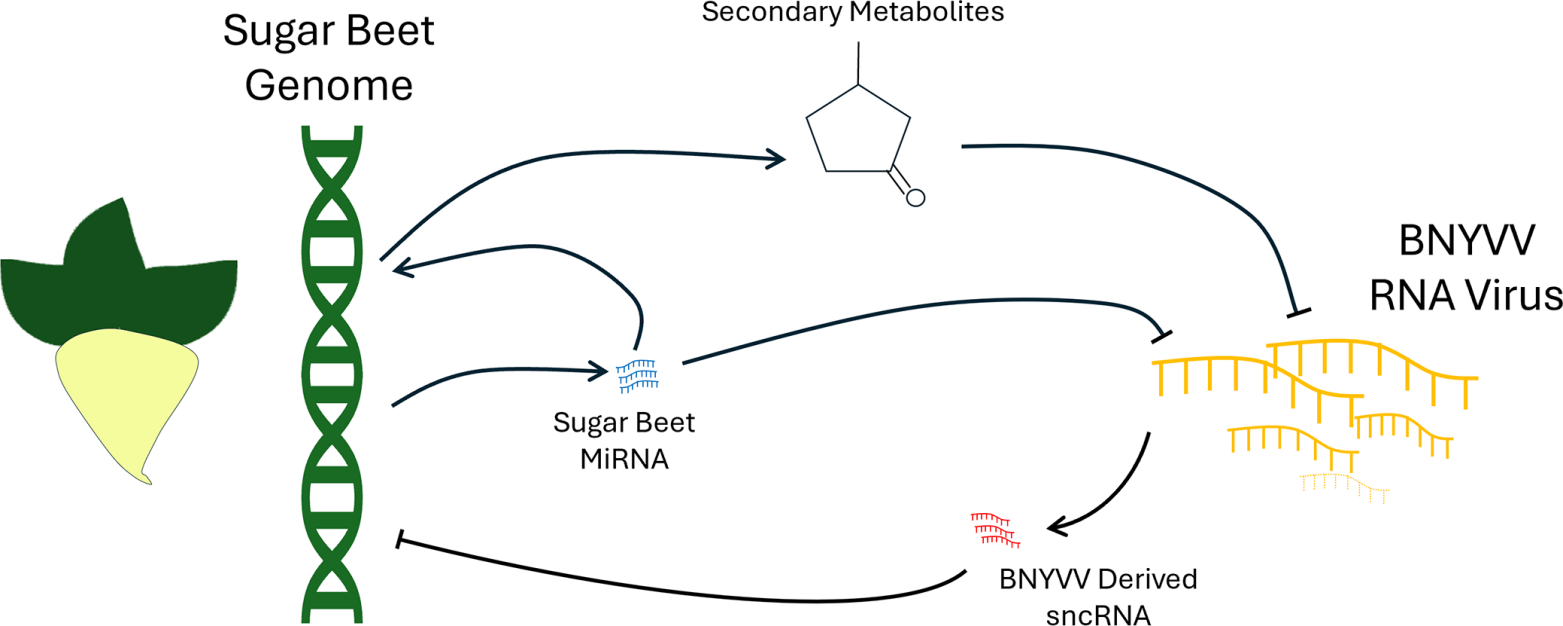
Proposed model of possible interaction between BNYVV and the sugar beet host via miRNA and sncRNA. This generalized model highlights possible actors responsible for both infection and resistance to that infection in the case of up-regulated miRNAs in the resistant line (KEMS12). While this simplified diagram omits complexities that are likely to exist, it highlights the potential roles of miRNA and sncRNA in virus–host interaction.

Our results point to multiple points of interaction (Fig. 8) that may play a role in both the pathogenicity of BNYVV and possible actors responsible that explain variation in disease resistance in our susceptible varieties. We observed differential expression of sugar beet miRNA with possible targets in both the BNYVV genome and the sugar beet genome. The gene targets in sugar beet showed enrichment for multiple metabolic pathways. We also observed sncRNA with strong evidence of viral origin that have sequence homology to regions of the sugar beet genome.

## Discussion

Disease resistance in plants is deployed at multiple levels including transcription, translation, post-transcriptional and translational modifications, metabolism and production of antimicrobial metabolites [39,40]. Sugar beet resistance in commercial cultivars against one of the most devastating diseases, rhizomania, across the world is primarily dependent upon the presence of dominant resistant (*Rz*) genes. We have previously found that KEMS09 contains a marker [41] associated (*R*^2^ ~0.09–0.37) with Rz1 resistance, KEMS12 shows some segregation for the marker and it is absent from KDH13. As we see strong resistance in viral load and phenotype (Fig. 1) in KEMS12 compared to KEMS09, the work presented here supports non-*Rz*-mediated resistance in the EMS mutant sugar beet breeding line, KEMS12. The primary goal of this work was to identify putative roles of sncRNAs, especially sugar beet-derived miRNAs, in resistance against BNYVV and elucidate the role of BNYVV-derived sncRNAs in viral pathogenicity. To dissect this, we used rhizomania-resistant and susceptible lines, different tissues, namely, roots and leaves from early (no visible symptoms) and late (major symptoms) infection stages, and comprehensive RNA sequencing approaches.

The DE miRNAs showed distinct regulation in the R line (vs. lines) depending upon tissue type (roots and leaves) and infection stages, early and late. Some examples include PC-5p-83783_281, PC-3p-81966_288, etc. (Fig. 3) that were up-regulated (no expression in the S lines) only in the roots and PC-5p-36370_644 that was down-regulated (very low expression) in the leaves at early infection stage in the R line, whereas examples of highly up-regulated (~10- to 16-fold) and down-regulated (~2.5-fold) miRNAs in the roots at late infection stage include PC-3p-2174_5149 and ath-miR162a-3p, respectively. Further evaluation of miRNA target genes in sugar beet shed light on the physiological and metabolic implications of such differential expressions of miRNAs leading to resistance or susceptibility. Some of the up-regulated or down-regulated genes in the R line observed in this study have been shown for their roles in resistance or susceptibility to viruses and other pathogens in plants. Down-regulation (two- to fourfold) of the MIP aquaporin candidate gene (*Bevul.2G122300*) targeted by PC-3p-81329_290 and PC-5p-42396_560 only in the leaves of the R line at late infection stage might indicate its role in leaf resistance as a negative regulator. The role of aquaporins in plants in the context of pathogen infections can act either way, contributing to resistance or susceptibility. This depends upon pathogen type, different isoforms of aquaporins, stages of infection, tissue types, etc. [42]. As an example, OsPIP2 in rice transports hydrogen peroxide (H_2_O_2_) to the cytoplasm and activates plant defence pathways against bacterial pathogens [43]. In another situation, during cucumber mosaic virus interaction with Arabidopsis, viral replication protein interacts with specific host aquaporins and aids in viral replication and spread [44]. Other sugar beet candidate genes such as zinc finger, potassium transporter and cysteine/methionine metabolism related showed a three to sixfold up-regulation only in the roots of the R line (vs. S) at late infection stage. This was due to down-regulation (~2- to 50-fold) of the miRNAs (ath-miR162a-3p, PC-5p-36370_644; Table 1) targeting these genes. Antiviral roles of zinc fingers have been implicated in translational repression and mRNA degradation in viruses and included antiviral defence mechanisms through their involvements in interferon signalling [45]. Engineered zinc fingers have been demonstrated to improve resistance against viruses in plants [46]. The role of cysteine in host plants’ defence responses against pathogens including viruses has been attributed to its level in the cytoplasm affecting the expression of genes associated with plant immunity, cross talk with salicylic acid signalling pathway and production of antiviral metabolites such as sulphur-containing compounds, phenolics, alkaloids, etc. [47]. Methionine cycle (MTC) and its associated pathways have been shown to play critical roles in antiviral defence responses in plants through the formation of MTC enzyme-multiprotein assemblies [48]. In rice, overexpression of the potassium transporter, OsHAK5, improved resistance against the rice grassy stunt virus through increased accumulation of reactive oxygen species and defence signalling [49]. The results taken together into consideration indicate key roles of miRNAs in sugar beet contributing to resistance against rhizomania.

The sncRNAs derived from BNYVV and their putative roles in pathogenicity during natural infection of sugar beet and development of disease symptoms were also investigated. Using genome alignment, a portion of the total sncRNA sequences derived from the BNYVV virus was characterized. Interestingly, the lengths of these BNYVV-derived sncRNA sequences were more defined and enriched for 21–22 bp (Fig. 5), compared to the larger distribution of sncRNAs. The virus-derived sncRNAs predominantly originated from RNA1 and RNA2 segments (Figs S4 and S5) and showed strong interactions with sugar beet genes (discussed later). The lack of RNA5-derived sncRNAs in our samples is consistent with earlier reports indicating that U.S. strains in general lack RNA5 segment [7]. The relative abundance of the BNYVV sncRNAs between genotypes is consistent with known resistance and susceptibility to BNYVV in these genotypes [25], with KEMS12 exhibiting highest resistance, KEMS09 exhibiting moderate susceptibility and KDH13 exhibiting high susceptibility. Although KDH13 was the most susceptible line and KEMS09 was the moderately susceptible line, at times, higher amounts of BNYVV-derived sncRNAs accumulated in KEMS09. Nevertheless, KEMS09 plants exhibited lesser stunting and some differences in mRNA expression vs. KDH13 (Fig. 1), indicating somewhat higher level of resistance in the former. Overall, viral load was much lower in the roots of KEMS12 (Fig. 1) compared to the susceptible lines and in general very low in the leaves when compared to the roots. The results presented here show clear interactions between the infection stages and genotypes regarding disease resistance, with higher abundance of BNYVV-derived sncRNAs at the early infection stage reflecting the phenotypic differences between genotypes more strongly than the late infection stage. Future studies in KEMS12 focusing on molecular events during very early stages of infection will capture early events, leading to resistance phenotype in this breeding line. Analysing the subset of BNYVV derived sncRNAs with high sequence complementarity to the sugar beet genome helped to dissect possible BNYVV-sugar beet interactions. The fraction of BNYVV sncRNAs that showed sequence complementarity with regions in the sugar beet genome featured a 100% match along 19–20 bp (Fig. 5), often having a 1–2 bp overhang. These sequences and alignment lengths are consistent with small interfering RNA (siRNA), where 2 nt of overhang is optimal for sequence-specific mRNA degradation [50]. The effect of the sncRNA complementarity to sugar beet genes is not clear while considering any specific gene (Fig. S6); however, the overall effect of higher accumulation of BNYVV sncRNAs was negatively correlated with lower expression of a subset of sugar beet genes (Fig. 7). The ambiguity of individual gene effects may be due to the smaller sample size of viral sncRNAs with relatively smaller number of reads for a specific sample. Future degradome analysis of BNYVV sncRNA target sugar beet genes will validate their precise roles in RNAi. The enrichment of MAP kinase activity, peptidyl-lysine trimethylation and transcription elongation functions among the 69 sugar beet genes highlights the potential pathways targeted by the virus. MAP kinase pathways are known to be important for plant defence signalling [51] and have been shown to be the targets of pathogen suppression by dephosphorylation [52] or blocking kinase activity [53].

In response to BNYVV-derived sncRNAs targeting key genes in sugar beet, a counter strategy involving cross-kingdom targeting of BNYVV genome by sugar beet was observed. Several sugar beet miRNAs (highly up-regulated in the R line) perfectly aligned to the BNYVV genome at the seed region of the miRNAs and other miRNAs showing perfect sequence complementarity are indicative of potential miRNA target sites (Table 4) in the viral genome. The known attributes of plant/host-derived miRNAs binding to the genome of RNA viruses [54] and the data presented here might shed light on additional strategies deployed by the R line in controlling BNYVV titre in the plants. In the majority of cases, sugar beet miRNAs putatively targeted genes associated with the core functions in the virus, namely, replication and transcription and, in addition, cell-to-cell movement. In several instances, multiple sugar beet miRNAs showed putative binding sites in the same viral genes at different locations that varied with leaves vs. roots and infection stages. Some examples included up-regulation (>4-fold in the R line vs. S) of aly-miR172b-5p in the leaves at early and late infection stages and targeted BNYVV RNA-dependent RNA polymerase, up-regulation (>4-fold in the R line vs. S) of ath-miR172c_1ss21GT in the roots at early and late infection stages and targeted BNYVV 42 kDa transport protein [55], etc.

We further investigated genome-wide distribution of putative sORFs in BNYVV, but it is unknown if these sORFs encode small peptides. RNA virus genome-derived small peptides and their role as pathogenicity factors have been demonstrated in other host systems [56]. Though numerous putative sORFs ranging between 7 and 30 aa were computationally detected across all RNA1-5 segments (Table S6), we were unable to detect any small peptides originating from these sORFs during natural infection of sugar beet roots. Interestingly, the peptides (ranging between 9 and 14 aa) that were detected in the roots at late infection stages originated from BNYVV 75 and 14 kDa proteins located in the RNA2 segment (Table 5, Fig. S4). The 75 kDa protein has been demonstrated to be essential for the transmission of BNYVV by the plasmodiophorid vector, *P. betae* [57], whereas the 14 kDa cysteine-rich protein (P14) has been attributed to viral silencing suppressor that counteracts host plant’s RNAi machinery [58]. The known functions of these two proteins in BNYVV and the observations from this study further raise the question of whether the processing of these longer peptides results in the production of smaller peptides which might augment their roles as pathogenicity factors and disease symptoms development. Processing larger peptides into smaller peptides in viruses which can play roles as pathogenicity factors has been reported in other studies [59]. Functional characterization of the BNYVV-derived small peptides in the future will determine their precise roles in viral pathogenicity. If proven, they may serve as potential targets in the virus to improve sugar beet resistance against rhizomania.

## Conclusion

The work presented here is the first comprehensive demonstration of putative roles of miRNAs and their target sugar beet genes involved in metabolic pathways contributing to rhizomania resistance in an EMS genetic background. We have mapped BNYVV-derived sncRNAs originating from different RNA segments in the virus (during natural infection in the field) and their putative targets in sugar beets in tissue-specific and infection stage-specific manners. Our work has identified key miRNAs in the R line that show putative target regions in the BNYVV genome. In addition, BNYVV-derived small peptides identified in this work may act as potential pathogenicity factors and will require further investigation to determine their precise roles in disease development. The information presented on sugar beet miRNAs and viral-derived sncRNAs and small peptides may serve as potential targets to improve sugar beet rhizomania resistance using cutting-edge functional genomic tools in the future.

## Supplementary material

10.1099/jgv.0.002193 Uncited Supplementary Material 1.

## Abbreviations

BNYVV: beet necrotic yellow vein virus
DE: differentially expressed
EMS: ethyl methanesulphonate
FPKM: fragments per kilobase of transcript per million mapped reads
GO: gene ontology
KEGG: Kyoto encyclopedia of genes and genomes
miRNA: microRNA
mRNAseq: mRNA sequencing
MTC: methionine cycle
R: resistant
S: susceptible
siRNA: small interfering RNA
sncRNA: small non-coding RNA
sORF: small ORF
sRNAseq: small RNA sequencing
