# Supplementary material for: Small RNA-mediated regulation of cross-kingdom gene expression in sugar beet genotypes resistant and susceptible to rhizomania
Source: J Gen Virol. 2025 Dec 17;106(12):002193. doi: 10.1099/jgv.0.002193 (PMC12710977; doi:10.1099/jgv.0.002193)
Supplement: Uncited Supplementary Material 1. [file jgv-106-02193-s001.pdf]

GO enrichment

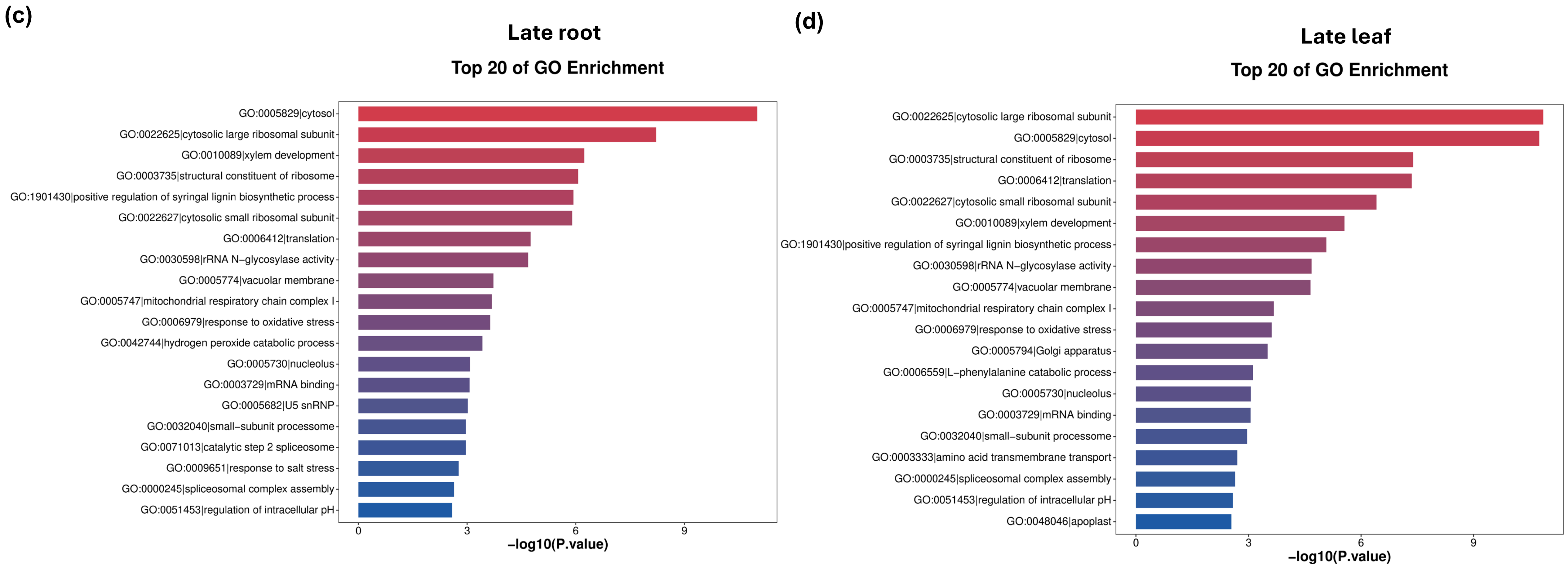

Fig. S1.

Gene ontology (GO) term of sugar beet target genes of differentially expressed (DE) sugar beet miRNAs at different stages of rhizomania infection. (a) Early root. (b) Early leaf. (c) Late root. (d) Late leaf. Data are Mean ± SE of 4 biological replicates (8 individual plants/replicate).

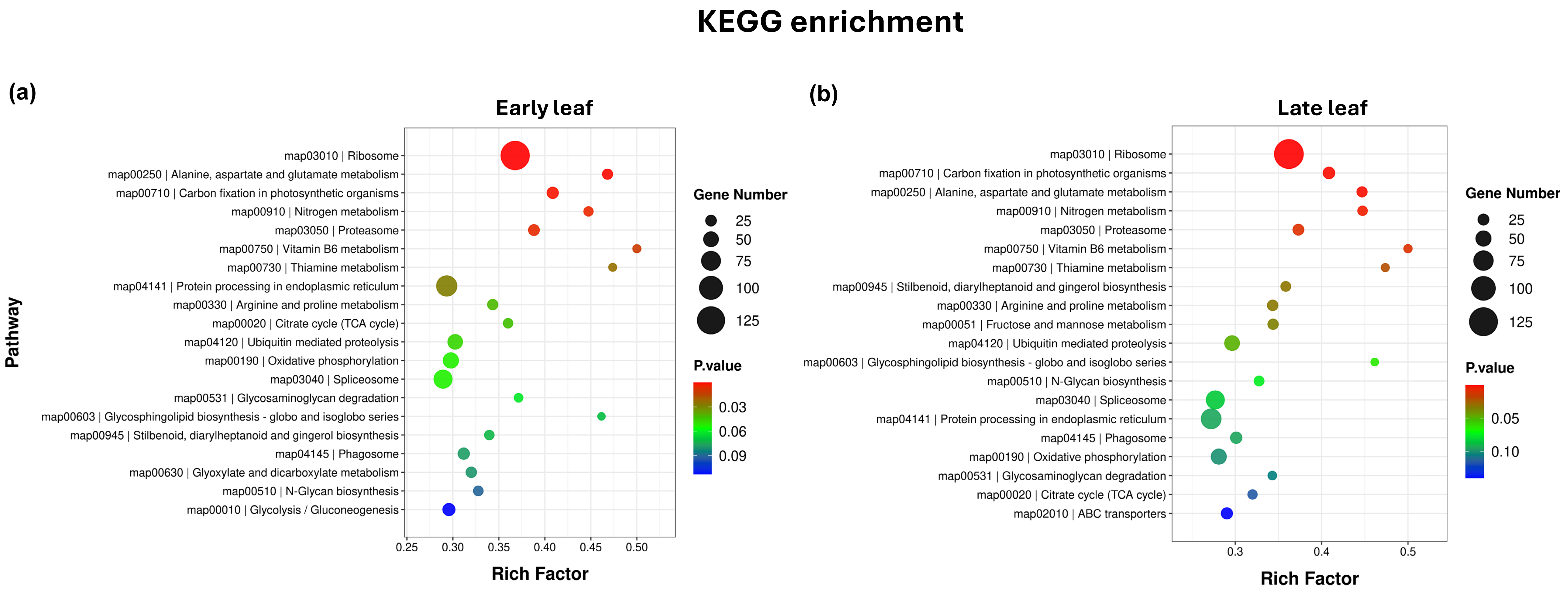

Fig. S2.

Kyoto Encyclopedia of Genes and Genomes (KEGG) enrichment of sugar beet target genes of differentially expressed (DE) sugar beet miRNAs at different stages of rhizomania infection. (a) Early leaf. (b) Late leaf. Data are Mean ± SE of 4 biological replicates (8 individual plants/replicate).

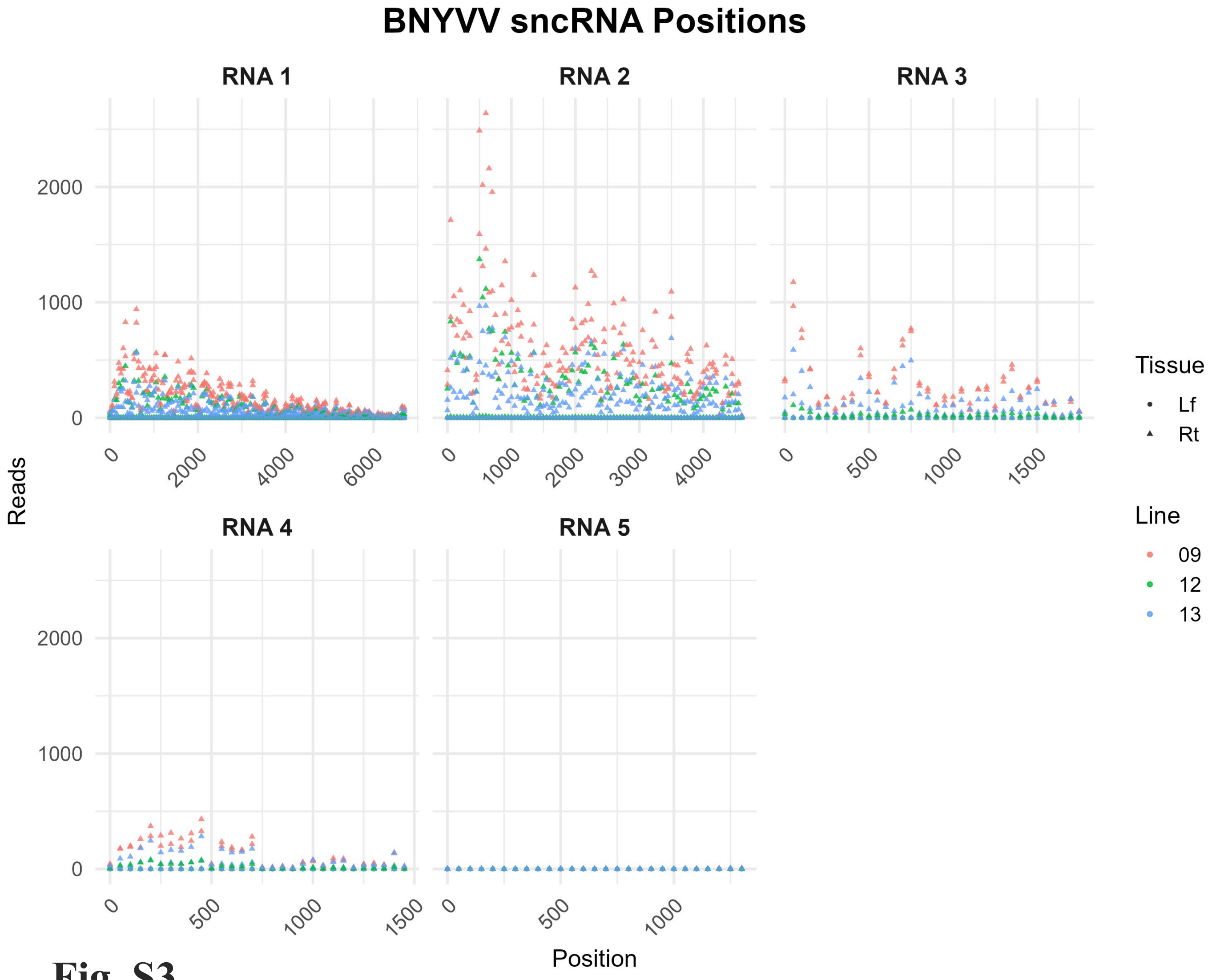

Fig. S3.

Locations of sncRNA that is putatively derived from the BNYVV virus. Tissue is designated by point shape, and sugar beet line is designated by color. X-axis designates base-pair position in the BNYVV RNA segments, and y-axis corresponds to the number of reads measured.

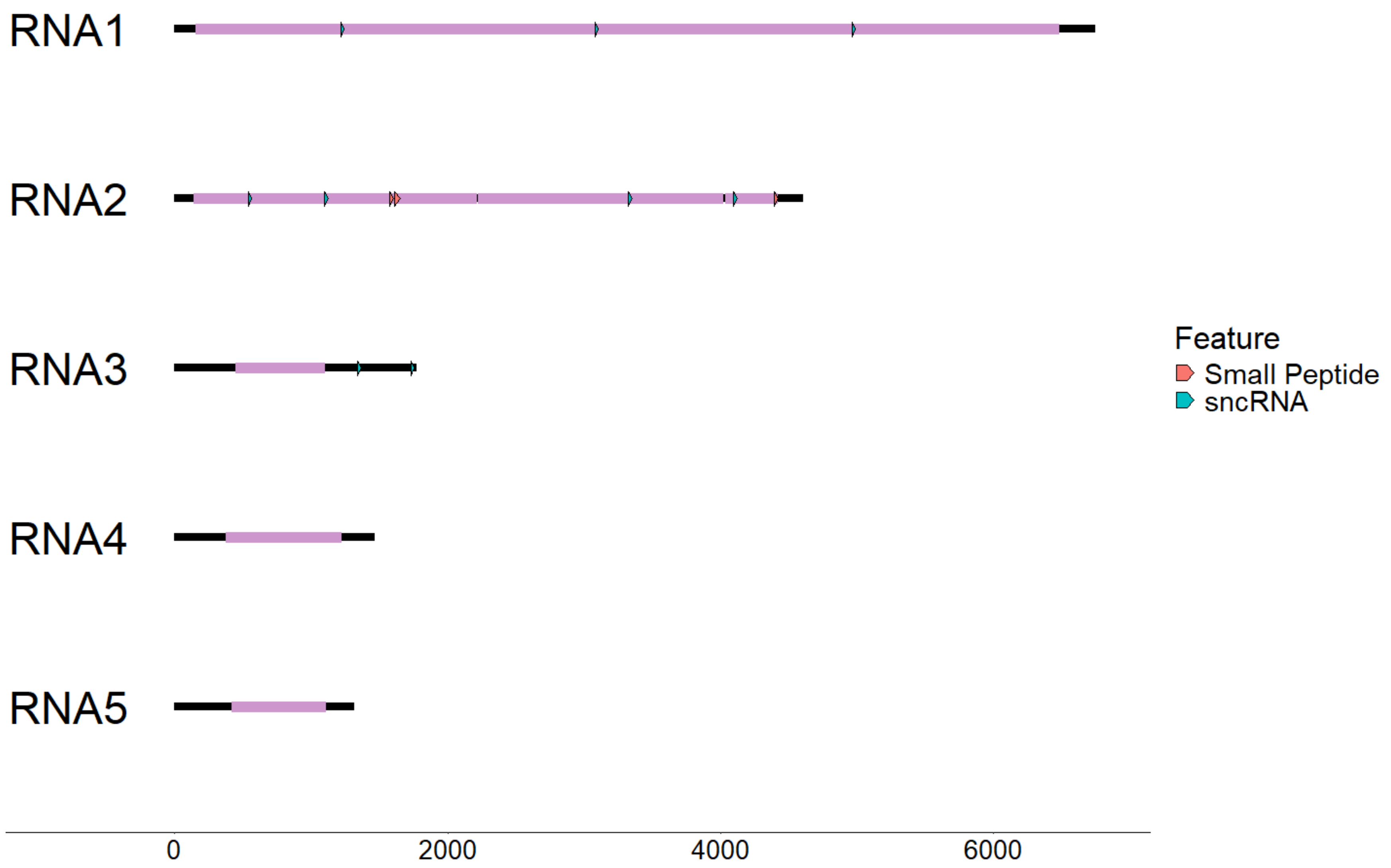

Fig. S4.

Diagram of BNYVV genome RNA segments. Small peptides and most highly expressed sncRNAs attributed to BNYVV are shown. Pink regions correspond to known protein coding genes.

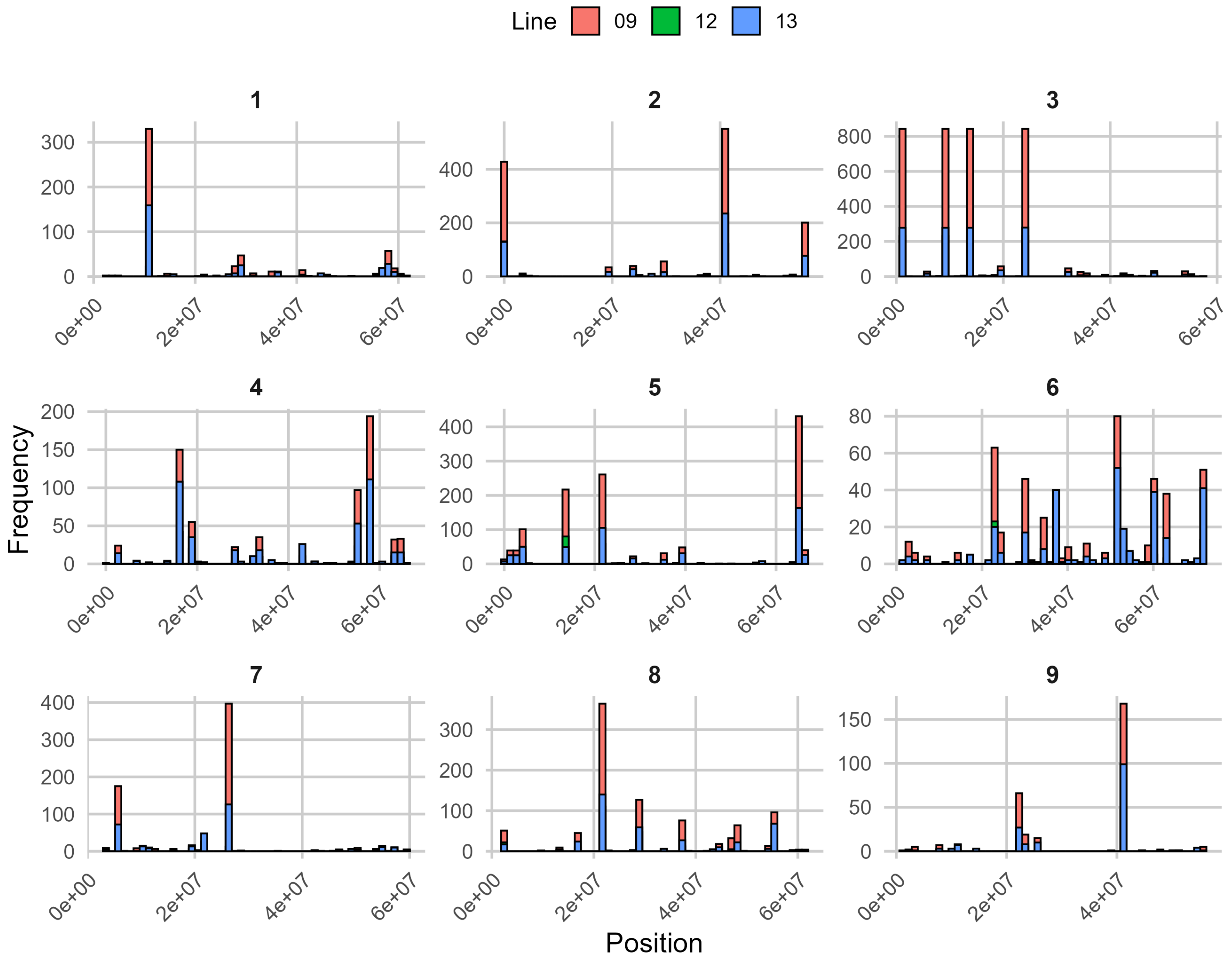

Fig. S5.

Mapping positions of putative BNYVV sncRNA against the EL10.2 sugar beet genome. Sugar beet line is designated by color. X-axis designates base-pair position along the 9 sugar bet chromosomes, and y-axis corresponds to the number of reads measured.

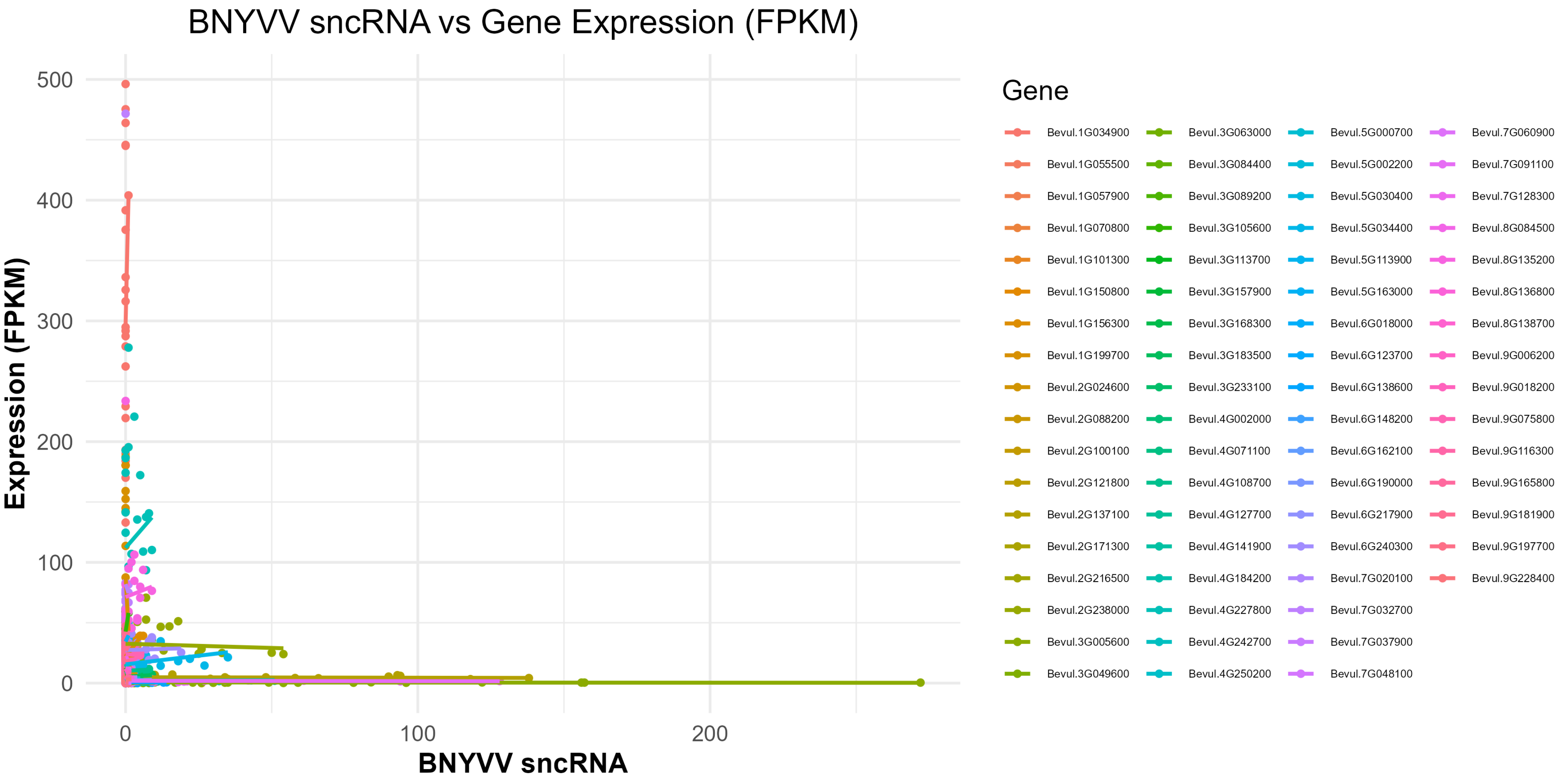

Fig. S6.

Scatterplot comparison for each gene with both mRNA expression (y-axis) and measured sncRNA putatively derived from BNYVV mapping against them. Linear correlations between are drawn for each gene, while none significantly show silencing action of sncRNAs from BNYVV.

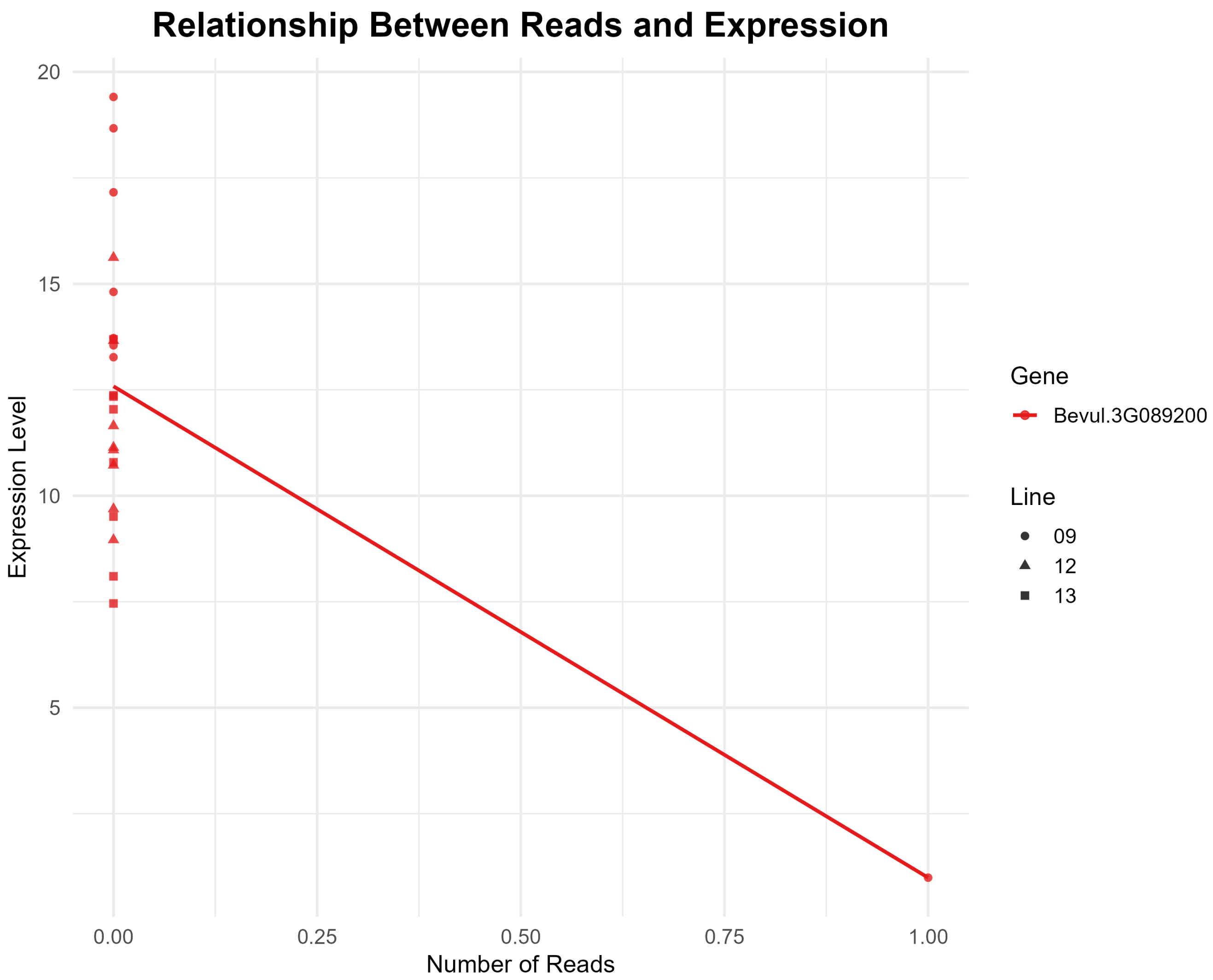

Fig. S7.

Scatterplot comparison for Bevul.3G089200 gene mRNA expression (y-axis) and measured sncRNA putatively derived from BNYVV mapping against them. The linear correlation between gene expression and BNYVV sncRNA is technically significant it is not meaningful.
